# Educational boundaries explain strength and variation in global fertility convergence

**DOI:** 10.1038/s41598-024-78735-2

**Published:** 2024-11-09

**Authors:** Hanbo Wu, Luca Maria Pesando

**Affiliations:** Division of Social Science, New York University Abu Dhabi, Saadiyat Campus, PO Box 129188, Abu Dhabi, United Arab Emirates

**Keywords:** Fertility, Convergence, Educational gradients, Human behaviour, Socioeconomic scenarios, Population dynamics

## Abstract

This paper shows that the level and timing of fertility are converging strongly over different measures of educational attainment using 65 years of data from 146 countries. Global convergence patterns are primarily driven by high-income societies, while sub-Saharan Africa is the world region that is converging most slowly, if not converging at all. Most importantly, levels of education matter heavily for explaining strength and variation in global fertility convergence, with two *intersecting educational gradients* suggesting: (i) stronger convergence over tertiary education followed, in turn, by secondary and primary; (ii) stronger convergence over education *completed* relative to education *attended*. Our findings provide important insights for addressing key challenges in global development and demography, and for informing policymakers as they evaluate the suitability of specific educational policies aimed at further narrowing inequalities between societies—such as supporting higher education as well as the successful completion of targeted educational cycles.

## Introduction

Since the 17 United Nations (UN) Sustainable Development Goals (SDGs) were agreed upon by the international community, there have been considerable concerns about priority setting for how to reach the best societal outcomes around the world. Among the various policy areas of intervention, education has been advocated as one of the most promising investments societies can make to ripen long-term benefits despite short-term costs^1^.

From a socio-demographic perspective, better education is associated with lower fertility and higher investments in children, lower mortality and better health, slower population growth, higher gender equality, and reduced forced migration^2^. As such, scholars and policymakers currently embrace education not only as a key component of SDGs, but also as a prerequisite for the achievement of other, broader, global development outcomes. Resonating with this idea, a highly influential study recently argued that most of the “population issues” faced by low-income countries, such as high fertility and rapid population growth, are in fact issues of human capital, defined as a combination of *schola et sanitate*, i.e., schooling and health^3^. Although appealing, this argument may benefit from additional empirical investigations leveraging global-level data and focused on the level and timing of fertility, key drivers of population growth.

This study explores whether changes in educational attainment underlie the way in which childbearing behaviour has been transforming over the past seven decades across 146 countries. Focusing on both fertility levels (*quantum*) and timing (*tempo*), our contribution to the existing literature is threefold. First, we bring the analysis of fertility convergence to a global level using growth-convergence techniques, updating previous efforts on the topic^4–7^. While demographic convergence—the idea that countries become increasingly similar to each other in some demographic domains—is not a goal per se, it is beneficial to the extent that it may be indicative of higher human capital investments, higher female labour force participation, and increased compatibility between family life and economic success, i.e., a series of outcomes that correlate with lower and later fertility^8,9^. Second, we assess convergence trends over different measures of educational attainment, rather than over time^5^ or over levels of development^10^. Third, we complement continuous measures of education such as years of schooling with an analysis of educational boundaries, namely primary, secondary, and tertiary, alongside an oft-neglected differentiation between education *attended* and education *completed*. This is a novel undertaking with the potential to shed light on “critical” educational thresholds that may be more or less conducive to convergence in demographic behaviour across societies. This can in turn inform the design and implementation of educational policies—not simply aiming at boosting school access and school enrolment—to be targeted to specific educational cycles.

## Materials and methods

### Measures of fertility and educational attainment

Fertility indicators for women were obtained from the 2022 update of the *World Population Prospects* (WPP), the official UN population estimates^11^. The WPP presents annual estimates of key demographic indicators, such as population size, life expectancy, and fertility, from 1950 to 2022 for 237 countries and areas.

We utilized three conventional measures of fertility provided by the WPP. First, we considered the total fertility rate (TFR), which is expressed as the average number of live births per woman in a given year. TFR is one of the most widely used demographic indicators that measures the level of fertility. As demographers suggested^12^, when studying fertility, one should pay attention to not only the quantum (i.e., “how many”) but also the tempo (i.e., “when”) of fertility, i.e., when births occur. As a result, we also looked at a second indicator, the mean age at childbearing (MACB), which is expressed as the average age of mothers at the birth of their children and has been widely used to assess timing of childbearing^13^. While this may not be the best indicator to capture timing of fertility as it does not properly capture “tempo effects” when cohort fertility is declining^12^, it is the most readily available measure in terms of country coverage and time span. Also, existing research has shown that in many contexts in which fertility has fallen below replacement, a rise in MACB may temporarily depress period fertility measures such as the TFR^14^, thus underscoring the importance of looking at the TFR and MACB jointly, especially when considering multiple countries at different stages of development and demographic transition^13^. An arguably superior indicator would be the mean age at first birth—capturing entry into parenthood—yet constructing a long time series of this indicator for multiple countries is challenging as it requires birth-order specific inputs (we nonetheless provide robustness checks on this indicator on a subsample). Lastly, we examined the net reproduction rate (NRR), an alternative measure of fertility that is not as commonly used as TFR and MACB. NRR is a generational measure capturing how many daughters are needed to replace mothers taking mortality conditions into account and is expressed as the average number of surviving daughters per mother. This is an oft-neglected valuable indicator, especially in global analyses that include low- and middle-income countries where child and adult mortality are still high^10^.

While extensive scholarship has delved into the interrelationships between fertility quantum and socioeconomic development^10,15^, why timing indicators such as MACB matter for socioeconomic development requires further elaboration. First, changes over time in MACB are the result of two intersecting forces. The first one is the decline in higher-order births occurring as countries move through their fertility transition. The second one is the change in the timing of births of specific orders, hence the net effect of these two forces varies among countries^13^. Second, in many countries with below-replacement fertility, strong social and economic forces exert upward pressure on MACB. Policy efforts in these same countries, such as increasing the efficiency of the educational system (or compressing the period of education), are underway to lower the age at which young women and men finish their secondary or tertiary education without lowering educational attainment or schooling quality^14^. These policies may represent “win-win” strategies addressing both individual health concerns—such as health risks associated with late pregnancies, as well as higher likelihood of involuntary childlessness—and demographic and economic aggregate concerns, such as declining fertility and population ageing^14^. Conversely, in countries with persistently high fertility, such as countries in sub-Saharan Africa (SSA), tempo policies work in the opposite direction, that is, aiming to increase MACB, potentially expanding the period devoted to human capital accumulation, in order to speed up fertility decline, hence lower the rate of population growth. Therefore, exploring convergence in this indicator in a cross-country comparative scenario is fundamental from a development perspective.

Data on education were obtained from the *Barro-Lee Educational Attainment Dataset*^16^, which collects information on educational attainment by gender and age from more than 600 censuses and surveys. We used the latest 2021 updated version of the Barro-Lee dataset (available at http://barrolee.com/?*p*=103). Education indicators in the Barro-Lee Dataset are available every five years between 1950 and 2015 and are available for 146 countries. All data and analyses in this study pertain to women, primarily due to more readily available time series data on women’s fertility across multiple countries.

We extracted various measures of educational attainment from the Barro-Lee Dataset. To measure a country’s overall level of educational attainment, we used the average years of schooling for that country’s female population aged 25–64. In addition to overall education, we also considered three critical educational thresholds, namely primary, secondary, and tertiary levels. To do so, we made use of the distribution of educational attainment in a country’s adult population. Specifically, we looked at the percentage of female population aged 25–64 in a country who *attended* primary, secondary, and tertiary education. To distinguish between incomplete and complete education, we also used the percentage of female population aged 25–64 who *completed* primary, secondary, and tertiary education. As the focus on ages 25–64 includes educational attainment of post-menopausal women (hence, “low” educational attainment of women 50–64 may pull down the level-specific educational composition values), we also ran several robustness checks using different age ranges.

### Statistical analysis

We linked annual fertility data from the WPP with quinquennial education data from the Barro-Lee Dataset, which results in an analytical sample of 146 countries, with fertility and education indicators observed every five years from 1950 to 2015 (balanced panel), providing over 2,000 country-year observations. The list of countries included in the analysis is presented in Supplementary Table S1.

We assessed fertility convergence over different measures of educational attainment by testing for $\:\beta\:$-convergence^17,18^, i.e., the catching-up of countries “lagging behind” in a specific indicator. $\:\beta\:$-convergence can be estimated with a traditional growth-convergence framework whereby the growth rate in an outcome over time is regressed on the base outcome, as per the following equation:

1 $$\:\frac{[\text{ln}\left({Y}_{i.t+n}\right)-\text{l}\text{n}\left({Y}_{i,t}\right)]}{n}=\alpha\:+\beta\:{Y}_{i,t}+{\epsilon\:}_{i}.\:\:\:\:\:\:\:\:\:\left(1\right)$$

Equation 1 has been vastly employed in the macroeconomic growth convergence literature^17–20^, and has also been used to assess cross-country convergence in fertility rates and other sociodemographic outcomes over time^5,21–23^. A negative sign on the $\:\beta\:$ coefficient indicates that lagging countries are catching up with leading countries (converging), while a positive sign means that laggards are falling farther behind (diverging). In terms of fertility levels, $\:\beta\:$-convergence occurs when the rate of decline in fertility in high-fertility countries is greater than the rate of decline in low-fertility countries. In terms of MACB, $\:\beta\:$-convergence occurs when the rate of increase in MACB among countries with low MACB is greater than the rate of increase among countries with high MACB.

In this study, we are interested in fertility convergence through a human capital lens, rather than convergence over time. Therefore, we made a simple adaptation to Eq. 1 by replacing the difference in time units, i.e., $\:n$ in Eq. 1, with the difference in another socioeconomic variable, in this case education. Specifically, we estimated the following equation:

2 $$\:\frac{[\text{ln}\left(FE{R}_{i,t+5}\right)-\text{ln}\left(FE{R}_{i,t}\right)]}{(ED{U}_{i,t+5}-ED{U}_{i,t})}=\alpha\:+\beta\:FE{R}_{i,t}+{\gamma\:}_{i}+{\epsilon\:}_{i},\:\:\:\:\:\:\:\:\:\left(2\right)$$

where $\:\text{ln}\left(FE{R}_{i,t+5}\right)-\text{ln}\left(FE{R}_{i,t}\right)$ is the change in a (natural logged) fertility indicator between years $\:t$ and $\:t+5$ for country $\:i$; and, accordingly, $\:ED{U}_{i,t+5}-ED{U}_{i,t}$ is the change in an education indicator over the same time period for the same country. The ratio on the left-hand side of Eq. 2 (changes in fertility over changes in education) was regressed on $\:FE{R}_{i,t}$, i.e., the fertility indicator in the base year $\:t$. In the model, $\:\alpha\:$ is the constant, $\:{\gamma\:}_{i}$ is the country fixed-effects controlling for within-country heterogeneity, and $\:{\epsilon\:}_{i}$ is the error term clustered at

the country level. With the addition of country fixed-effects, our model is assuming different intercepts for each country, yet common speed of convergence (i.e., same slope). Note that a similar adaptation of the growth-convergence equation was made in a former study using the difference in Human Development Index (HDI) as denominator^10^.

The coefficient of interest is $\:\beta\:$ in Eq. 2. A negative $\:\beta\:$ indicates convergence—countries becoming increasingly similar to each other as educational attainment increases, while a positive $\:\beta\:$ indicates divergence—countries becoming increasingly dissimilar to each other as educational attainment increases, and a null $\:\beta\:$ indicates persistent differences (neither convergence nor divergence). While sign is key in this context, magnitudes matter too, as they measure “speed” of convergence; ultimately, this is a growth rate, hence the estimated coefficient provides the association between a base level in a fertility indicator and the growth rate of that same indicator over measures of education. To facilitate comparisons across differently scaled outcomes, we present standardized coefficients throughout this paper.

As the computation of growth rates over education may yield extreme values due to small changes in educational attainment across years, we excluded outliers falling outside the first quartile minus three times the interquartile range (IQR), and the third quartile plus three times the IQR.

It is important to stress that growth-convergence techniques are not meant to estimate causal relationships—in this case between educational change and fertility change—but to identify empirical regularities describing whether specific variables become increasingly similar or dissimilar to each other across units (countries, here) as time goes by (or as education changes, here).

## Results

### Descriptive findings

**Fig. 1:**
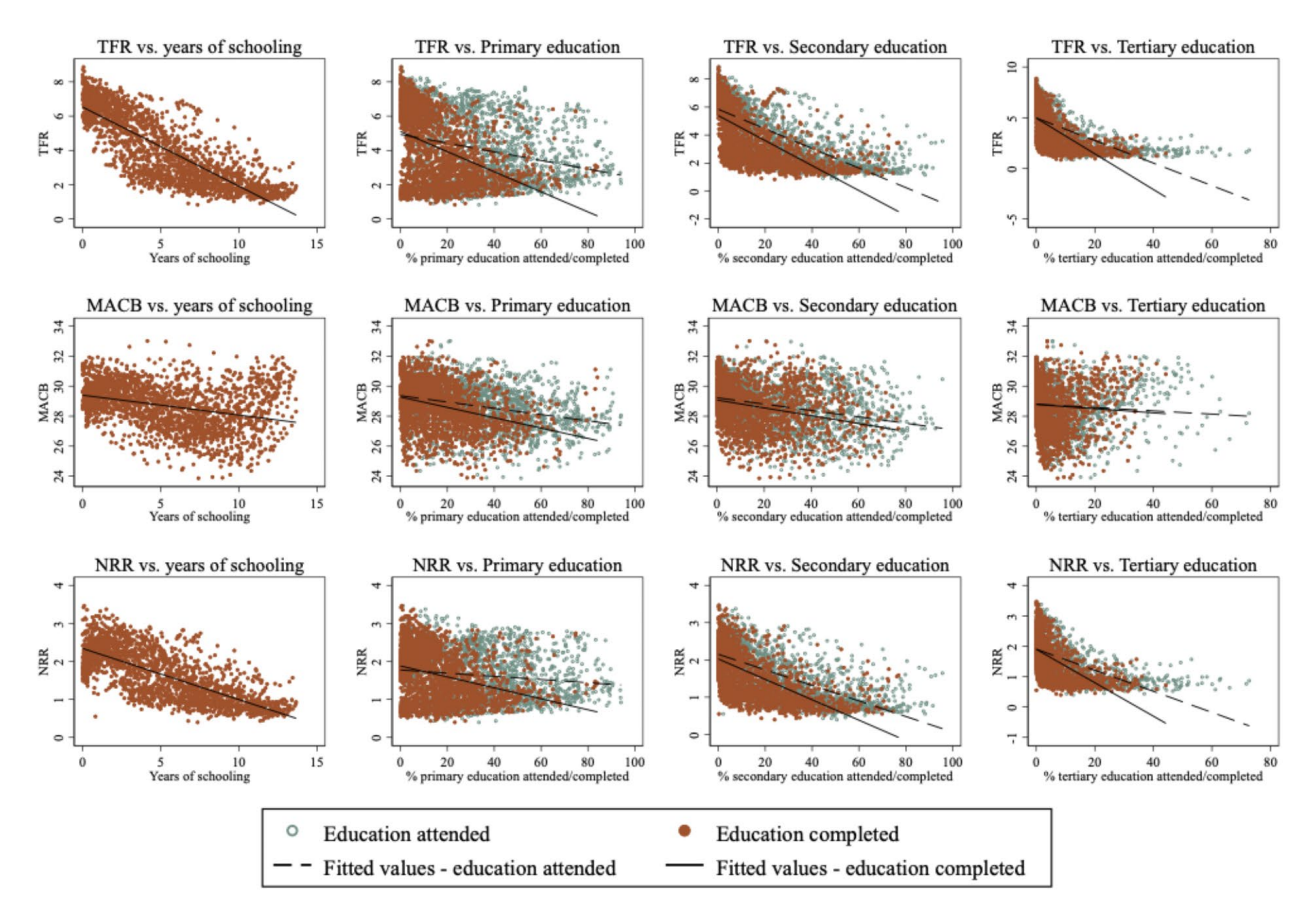
Scatter plots showing associations between fertility indicators and different measures of educational attainment. All country-year observations pooled. Rows report fertility indicators (top: TFR; middle: MACB; bottom: NRR) and columns report education indicators.

Before presenting results of the convergence analysis, we plot each fertility indicator against each measure of educational attainment in Fig. 1 (first row: TFR; second row: MACB; third row: NRR), which shows clear negative associations between fertility measures and education. Associations are more negative as levels of education increase (except for MACB, showing evidence of non-linearities) and when completed education (rather than attended) is considered. While informative, these associations do not convey whether countries have become increasingly similar (or dissimilar) to each other on these fertility indicators as education expands. Therefore, we next present convergence analyses.

### Estimates of β-convergence coefficients

**Fig. 2:**
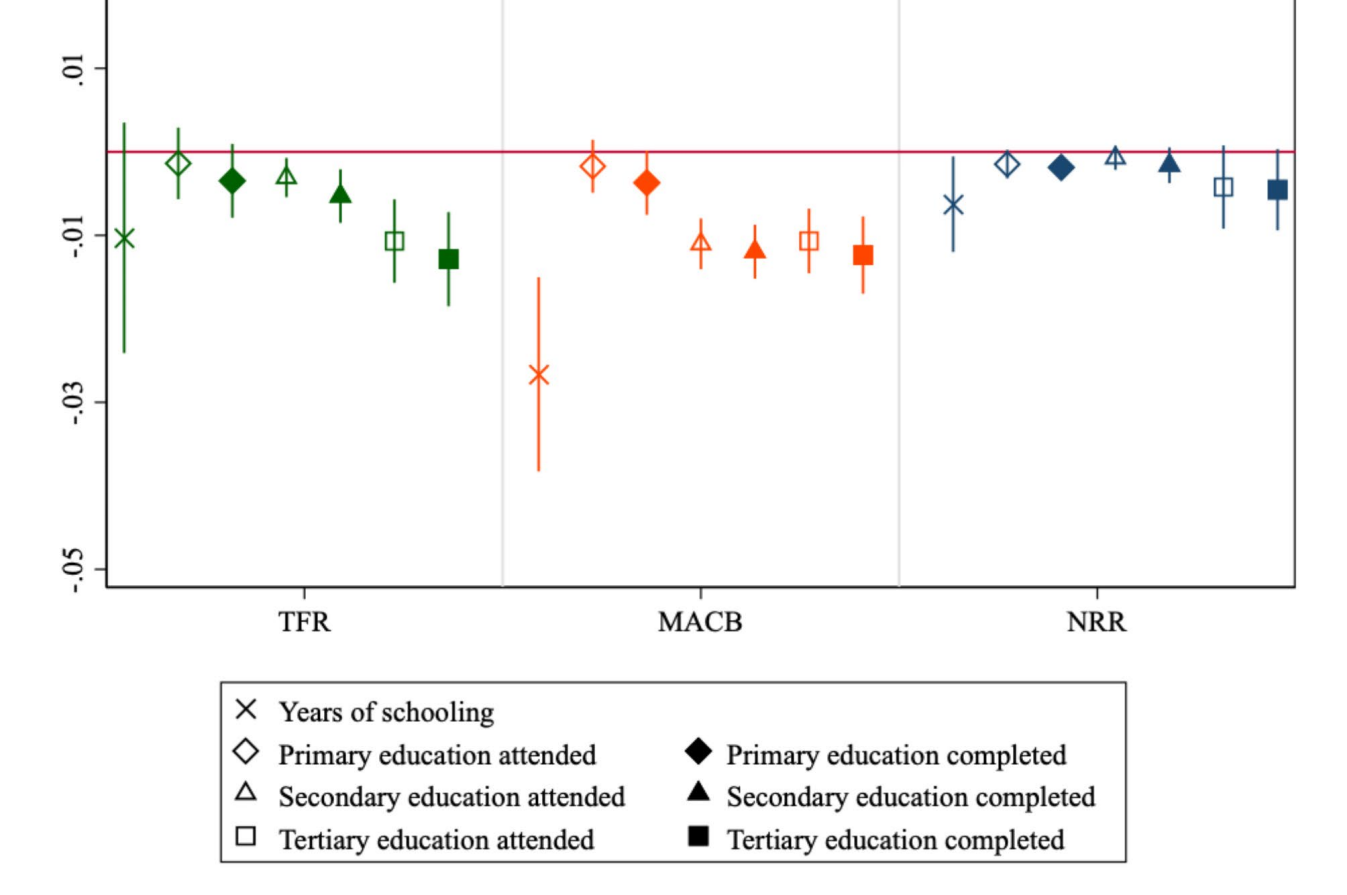
*β*-convergence standardized coefficients in fertility over educational attainment, all 146 countries. 95% confidence intervals reported.

Figure 2 reports $\:\beta\:$-convergence coefficients estimated using Eq. 2 for all 146 countries and all years pooled. In this figure and other figures showing estimated $\:\beta\:$-coefficients, different symbols indicate different education measures: × = years of schooling; ♢= primary education; △= secondary education; □ = tertiary education; hollow markers = education attended; filled markers = education completed. All $\:\beta\:$ coefficients have negative sign, providing evidence of global fertility convergence over education measured through both years of schooling and educational thresholds. With respect to specific indicators, convergence in TFR and MACB is more pronounced than that in NRR in terms of both effect size and statistical significance, likely due to inconclusive evidence on mortality convergence^24^, accompanied by widening international health inequalities^25^. To provide an interpretation of one coefficient, we observe that a one-year increase in “base” MACB is associated with roughly a 2.7% reduction in the growth rate of MACB over years of schooling. Most importantly, there is evidence of neat educational attainment gradients whereby the effect size of $\:\beta\:$-convergence coefficients is greater (i.e., more negative) as the higher the level of education achieved (tertiary > secondary > primary), particularly for TFR and MACB. Not least, within each level, completion of education (filled markers) contributes more to fertility convergence than just attendance (hollow markers). Thus, our findings underscore two intersecting educational gradients, one tied to educational thresholds, and one tied to attending versus completing educational cycles within each threshold.

**Fig. 3:**
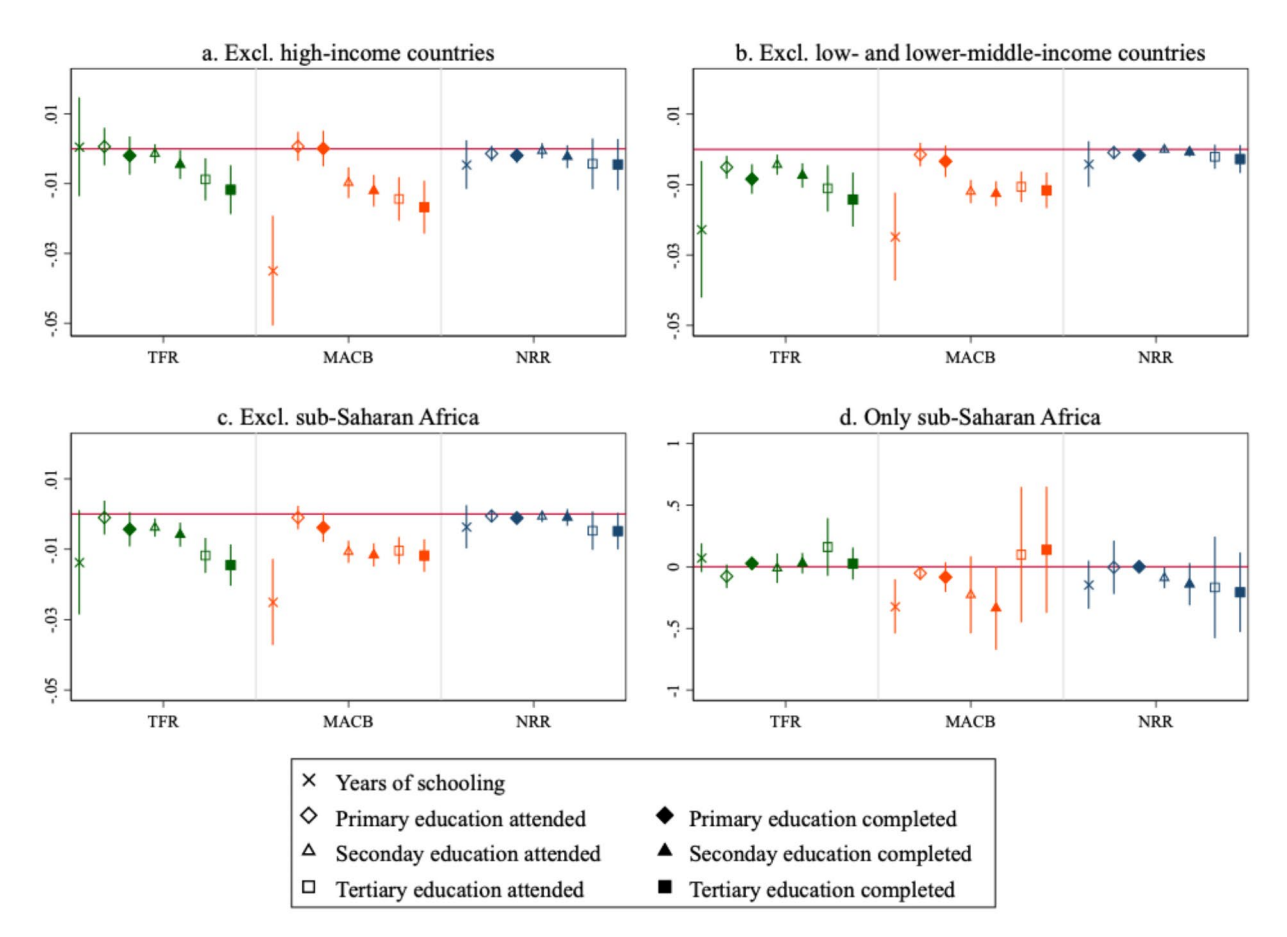
*β*-convergence standardized coefficients in fertility over educational attainment, subregional analyses: (**a**) excluding high-income countries (54 countries); (**b**) excluding low- and lower-middle-income countries (55 countries); (**c**) excluding sub-Saharan African countries (32 countries); (**d**) keeping sub-Saharan African only (note that this last panel is on a different scale). 95% confidence intervals reported. Note that the y-axis in Panel d is on a different scale, to accommodate wider confidence intervals.

We next report results of subregional analyses in Fig. 3. In line with previous research^5,10^, these subregional analyses were conducted by excluding selected countries from the sample in order to preserve sample variability by retaining adequate sample sizes. Excluding high-income countries (Fig. 3a) makes convergence in TFR over educational attainment weaker, with coefficients turning positive (yet not statistically significant, *P* = 0.936) for changes in TFR over changes in years of schooling. Evidence of convergence in both TFR and MACB over primary education weakens substantially. Conversely, excluding low- and lower-middle-income countries (Fig. 3b) makes convergence in TFR over all measures of education—in particular, over primary and secondary education completed—stronger both in magnitude and statistical significance. Findings on MACB and NRR are generally consistent with all-sample analyses, despite wider error bars due to smaller sample sizes. Excluding SSA countries (Fig. 3c) provides a figure that closely mirrors Fig. 3b. This is reasonable, as around 70% of countries in the low- and lower-middle-income group are in SSA. Excluding SSA, we also observe that convergence in NRR over tertiary education (foremost, completed education) is stronger, which is consistent with the idea that, once poorest countries are excluded, TFR and NRR are increasingly similar to each other, as mortality at young and adult ages is low. Lastly, Fig. 3.d displays model estimates limited to SSA, confirming widely different patterns in this world region: no evidence of convergence is observed for TFR. If anything, estimates for TFR are positive in SSA, especially over tertiary education. The same result is observed for convergence in MACB over tertiary education, although error bars are wide given the low prevalence of tertiary education in most parts of the region.

### Robustness checks

We recognize that other factors may affect fertility, yet we intentionally kept the convergence model simple to isolate the role of education (acknowledging that many other variables may co-vary with education). For example, availability of contraception is closely related to fertility and has increased dramatically over the study period. As a robustness check, we reran Eq. 2 with a covariate controlling for a country’s contraceptive prevalence (modern methods) among married women in a given year. This control variable was extracted from the UN *Family Planning Indicators* 2022 dataset^26^, which provides annual estimates of key family planning indicators from 1970 to the present for 197 countries. It should be noted that because data on contraception is available for a shorter period of time and is sparser in some countries, both the number of countries and the number of country-year observations reduce as compared to the main analysis (134 countries, about 700 country-year observations).

**Fig. 4:**
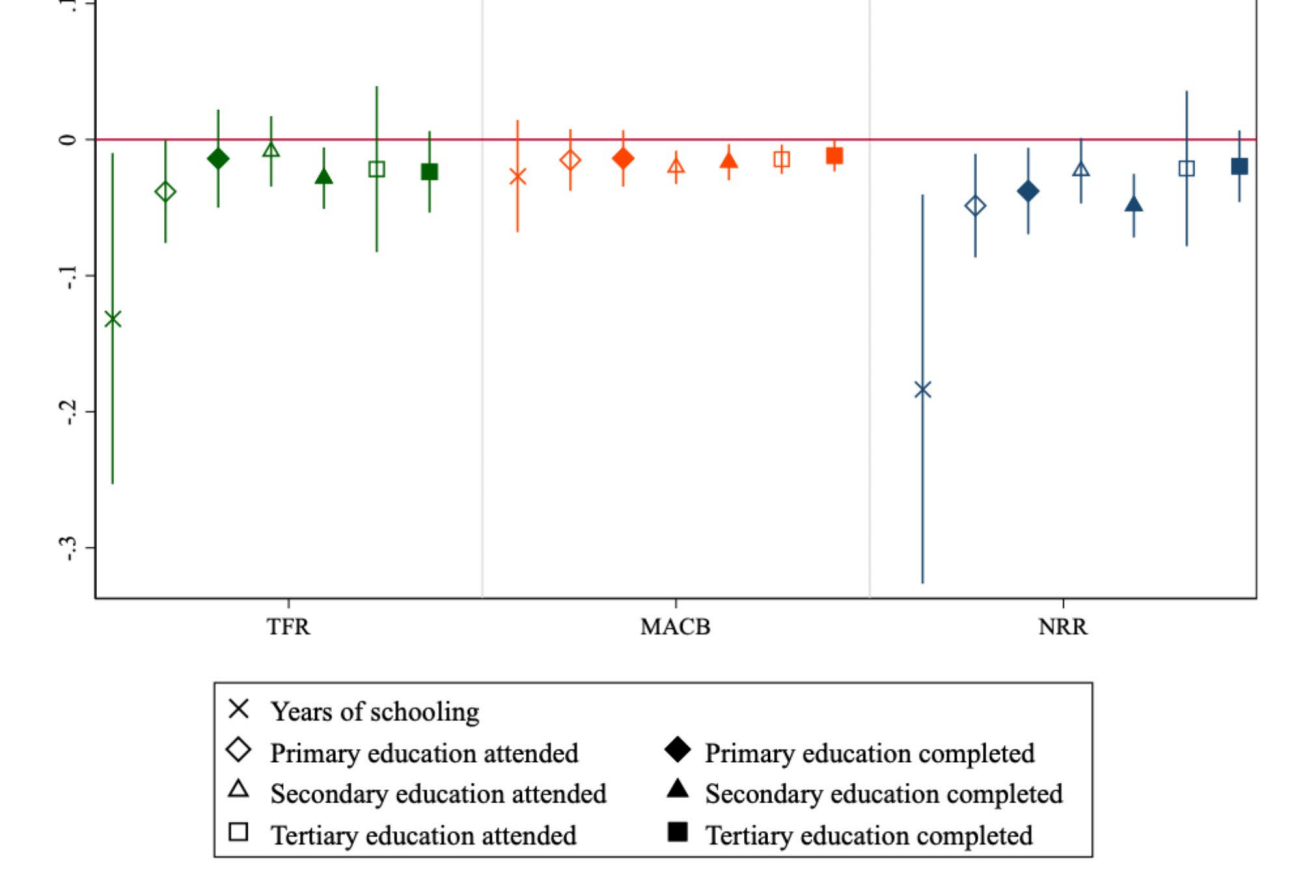
*β*-convergence standardized coefficients in fertility over educational attainment controlling for contraceptive prevalence (134 countries). 95% confidence intervals reported. Note that the y-axis in this Figure is on a different scale relative to Fig. 2, to accommodate wider confidence intervals. This does not imply lower magnitudes of* β*-convergence coefficients; actually, the opposite is true.

Estimates of* β*-convergence coefficients after controlling for contraceptive prevalence are depicted in Fig. 4. Results are largely robust and consistent: adding contraceptive prevalence as a covariate in Eq. 2 does not remove evidence of* β*-convergence in fertility indicators. Quite the opposite, convergence coefficients tend to be larger in magnitude (although confidence intervals are too)—in this respect, note that Figs. 2 and 4 are on a different y-axis. Convergence in NRR is even more evident after controlling for contraception. Evidence on gradients is weaker, though, likely due to presence of educational gradients in contraceptive use—yet another reason why we want to keep the main model as simple as possible.

Demographic data from the WPP provide the most comprehensive data available for social scientists interested in conducting global-level analyses that include as many countries as possible. Nonetheless, we acknowledge that for some countries fertility indicators in the WPP are modelled in the presence of sparse raw data. To address potential concerns about modelling (on education, mostly) underlying the WPP fertility indicators, we reran Eq. 2 with a much smaller sample of countries and country-year observations, using data on raw fertility (for simplicity, we only consider TFR) from the *Demographic and Health Surveys* (DHS) STATcompiler^27^. Note that this reduces the sample to about 15% of the original one, covering approximately 54 countries and 150 country-year observations.

Supplementary Fig. S1a shows results using DHS data. We fail to find evidence of fertility convergence in this sample of countries, and in fact the positive sign on some coefficients indicates increasing dissimilarity between countries. The results are generally consistent with Fig. 3b and d, as DHS data were collected in low- and middle-income countries only, with an over-representation of countries in SSA. In addition to directly using DHS data, we replicated this same analysis using WPP data but on a subset of countries and country-year observations identical to the DHS sample. Results, presented in Fig. S1b, also indicate a lack of statistically significant evidence of fertility convergence in this subset of countries.

Next, as anticipated above, we conducted a robustness check replacing the MACB with the mean age at first birth, available for a subsample of countries (about 100 countries and 400 country-year observations, because for the majority of countries data on mean age at first birth are only available after 2000). We pooled data from two different sources: the Global Data Lab for low- and middle-income countries^28^ and the UNECE Statistical Database for European and North American Countries^29^. We then re-estimated the convergence equation with this more proximate measure of the timing of entry into motherhood and provided results in Supplementary Fig. S2.

Compared with results on MACB (Fig. 2), the convergence coefficient in mean age at first birth over years of schooling is not significantly different from zero, and evidence of educational gradients weaken, driven by the lack of convergence on primary education. Nonetheless, in line with the main analyses, we document strong convergence over secondary and tertiary education, and even more so when these cycles are completed (rather than attended). As such, our overall conclusion that convergence exists, and is primarily driven by expansion of higher education holds irrespective of whether we focus on MACB or age at first birth.

Lastly, we ran robustness checks varying the age ranges of education indicators (Fig. S3), which show essentially identical results and educational gradients.

## Discussion

In light of the massive educational expansion and dramatic changes in fertility that the world is undergoing, this study contributed to existing scholarship by examining global convergence patterns in fertility indicators over a key dimension of global development, namely female educational attainment. This is a novel endeavour, aligned with the idea that countries are increasingly converging towards similar levels of educational attainment, hence suggesting a “convergence (in demographic indicators) within convergence (in education indicators)” dynamics^30^. We found that, over the past 65 years, fertility converged strongly over different measures of educational attainment, and was primarily driven by high-income societies, at least as far as TFR is concerned. We also showed that countries within SSA are the ones that converged most slowly, if not converging at all, thus exerting a “braking effect” on the global* β*-convergence coefficient. This finding aligns with prior research on demographic convergence over time^5^ and over levels of human development^10^, suggesting that regional convergence trends would depart less from global trends in the absence of SSA. Overall, we observed consistent convergence trends for indicators such as TFR and MACB, revealing convergence in both levels and timing of fertility, yet less so in generational indicators such as the NRR, a frequently overlooked indicator which, by taking women’s survival and sex ratios at birth into account, is of fundamental relevance for the study of global demographic dynamics, particularly across low- and middle-income countries where infant mortality remains a pressing concern. Weaker evidence of convergence in NRR hints at complex interplays between population dynamics whereby fertility, mortality and, in few countries, sex-ratio distortions may be pulling the convergence coefficient in different directions^24,25^. Given the inconclusive evidence, we believe exploring convergence dynamics in generational indicators is a valuable area for further research.

On top of global patterns by years of schooling, we offered new evidence on strength and variation in fertility convergence across educational boundaries, discussing two *intersecting educational gradients* suggesting stronger convergence over tertiary education followed, in turn, by secondary and primary, alongside stronger convergence over education completed relative to education attended. Our study is thus unique in uncovering that global convergence patterns do mask fundamental heterogeneity pointing to the growing importance of secondary and tertiary education—relative to primary—alongside the crucial need to shift the policy focus from school attendance to successful school-cycle completion.

Open questions remain on the extent to which similar convergence trends would be observed over indicators of educational “quality” instead of quantity, as growing evidence suggests that massive educational expansion has often come at the expense of quality^31,32^. Addressing such a question leveraging time-series on indicators of educational quality—such as, for instance, literacy rates, enrolment in public vs. private schools, or more sophisticated measures such as skills in literacy adjusted mean years of schooling^32^—would greatly enrich the sociological literature on horizontal inequalities in education, as well as closely match development policy priorities increasingly focused on quality over quantity. Similarly, a valuable extension of this work may consider focusing on male fertility measures, increasingly popular in socio-demographic studies^33^, as well as male education indicators.

All in all, our findings provide important insights for addressing key challenges in global development and demography, and for informing policymakers as they evaluate the suitability of specific educational policies aimed at further narrowing inequalities between societies, such as supporting higher education as well as the completion of targeted educational cycles. Educational gradients are observed across all countries, and stronger evidence of convergence in fertility over education completed (relative to attended) aligns with—and provides further validation to—global social efforts and investments that target ensuring that children and adolescents not only have access to school and attend regularly, but also reach the completion of their educational cycles. In light of these findings, there is reason and hope to believe that further advances in dimensions such as literacy and education—and, particularly, advances in tertiary education, especially within SSA—might contribute to reducing disparities across world regions and place the low-income world on a more defined convergence trajectory toward lower and later fertility.

## Electronic supplementary material

Below is the link to the electronic supplementary material.

Supplementary Material 1

## Data Availability

The data underlying this article are publicly available at https://population.un.org/wpp/ and at http://barrolee.com/. The datasets used and/or analysed during the current study available from the corresponding author on reasonable request.
