# Supplementary material for: Educational boundaries explain strength and variation in global fertility convergence
Source: Sci Rep. 2024 Nov 9;14:27323. doi: 10.1038/s41598-024-78735-2 (PMC11550852; doi:10.1038/s41598-024-78735-2)

Educational boundaries explain strength and variation in global fertility convergence

Hanbo Wu[1,*] and Luca Maria Pesando[1]

[1]Division of Social Science, New York University Abu Dhabi, Saadiyat Campus, PO Box 29188, Abu Dhabi, United Arab Emirates

[*]hanbo.wu@nyu.edu

Supplementary information

Table S1. List of countries

Subregions	Countries
Europe & North America	Albania; Austria; Belgium; Bulgaria; Canada; Croatia; Czechia; Denmark; Estonia; Finland; France; Germany; Greece; Hungary; Iceland; Ireland; Italy; Latvia; Lithuania; Luxembourg; Malta; Netherlands; Norway; Poland; Portugal; Republic of Moldova; Romania; Russia; Serbia; Slovakia; Slovenia; Spain; Sweden; Switzerland; Ukraine; United Kingdom; United States of America
Sub-Saharan Africa	Benin; Botswana; Burundi; Cameroon; Central African Republic; Congo; Côte d'Ivoire; Democratic Republic of the Congo; Eswatini; Gabon; Gambia; Ghana; Kenya; Lesotho; Liberia; Malawi; Mali; Mauritania; Mauritius; Mozambique; Namibia; Niger; Réunion; Rwanda; Senegal; Sierra Leone; South Africa; Togo; Uganda; United Republic of Tanzania; Zambia; Zimbabwe
North Africa & West Asia	Algeria; Armenia; Bahrain; Cyprus; Egypt; Iraq; Israel; Jordan; Kuwait; Libya; Morocco; Qatar; Saudi Arabia; Sudan; Syrian Arab Republic; Tunisia; Türkiye; United Arab Emirates; Yemen
South & Central Asia	Afghanistan; Bangladesh; India; Iran; Kazakhstan; Kyrgyzstan; Maldives; Nepal; Pakistan; Sri Lanka; Tajikistan
East & Southeast Asia	Brunei Darussalam; Cambodia; China; China, Hong Kong SAR; China, Macao SAR; Indonesia; Japan; Lao People's Democratic Republic; Malaysia; Mongolia; Myanmar; Philippines; Republic of Korea; Singapore; Taiwan; Thailand; Viet Nam
Latin America & the Caribbean	Argentina; Barbados; Belize; Bolivia; Brazil; Chile; Colombia; Costa Rica; Cuba; Dominican Republic; Ecuador; El Salvador; Guatemala; Guyana; Haiti; Honduras; Jamaica; Mexico; Nicaragua; Panama; Paraguay; Peru; Trinidad and Tobago; Uruguay; Venezuela
Other	Australia; Fiji; New Zealand; Papua New Guinea; Tonga

Figure S1. β-convergence standardized coefficients in total fertility rate (TFR) over educational attainment, fertility measure from (a) the Demographic and Health Surveys; (b) World Population Prospects; same countries included. 95% confidence intervals reported.

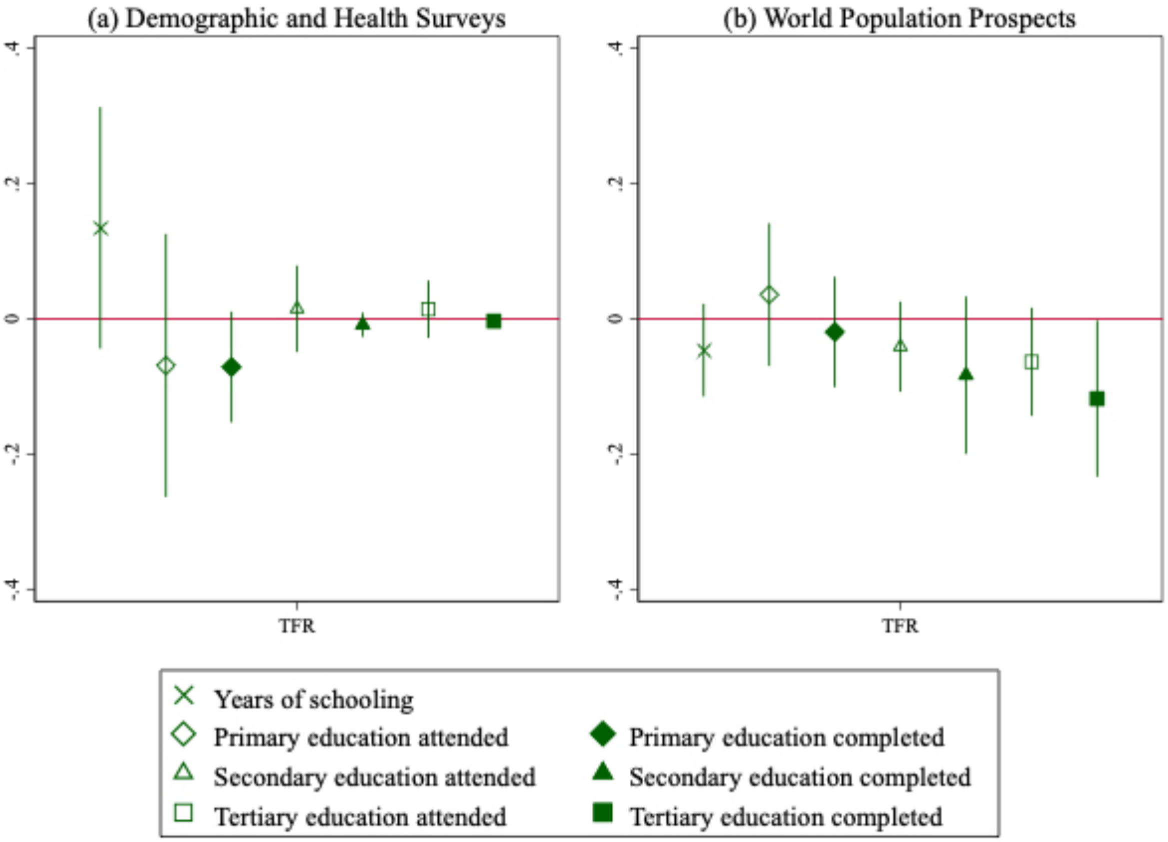

Figure S2. β-convergence standardized coefficients in women's mean age at first birth over educational attainment, data from the Global Data Lab and the UNECE Statistical Database. 95% confidence intervals reported.

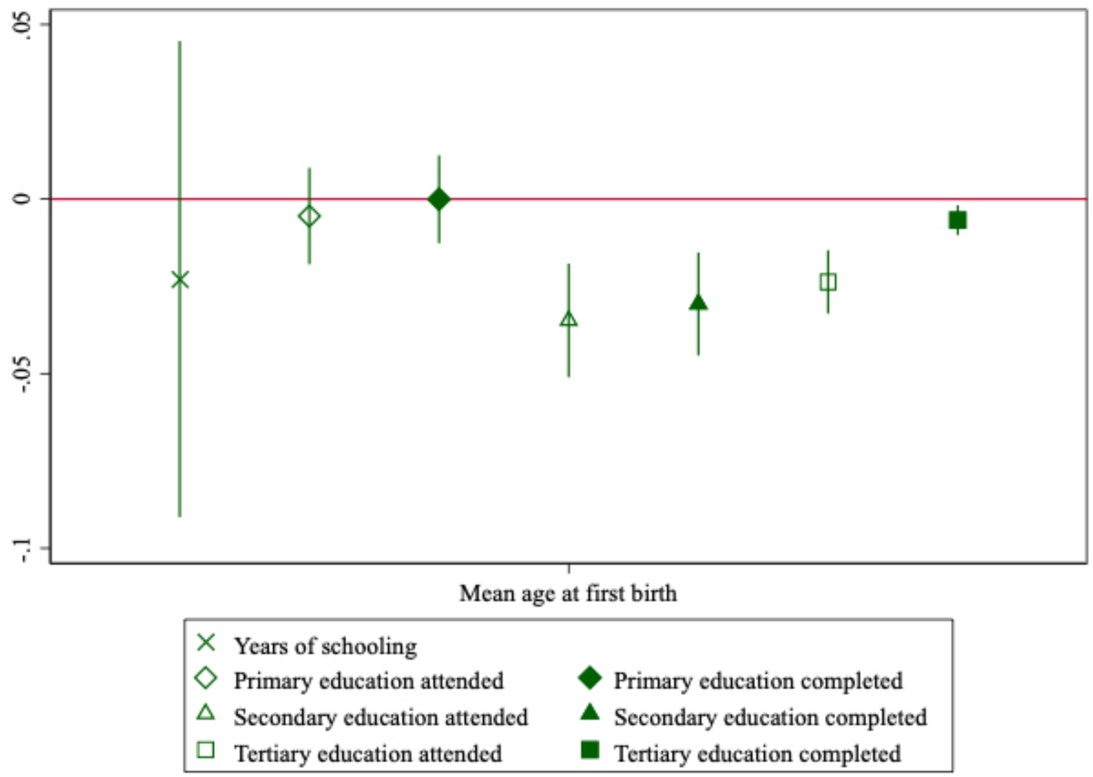

Figure S3. β-convergence standardized coefficients in fertility over educational attainment for women aged (a) 25–34; (b) 35–44; (c) 45–54; (d) 55–64. 95% confidence intervals reported.

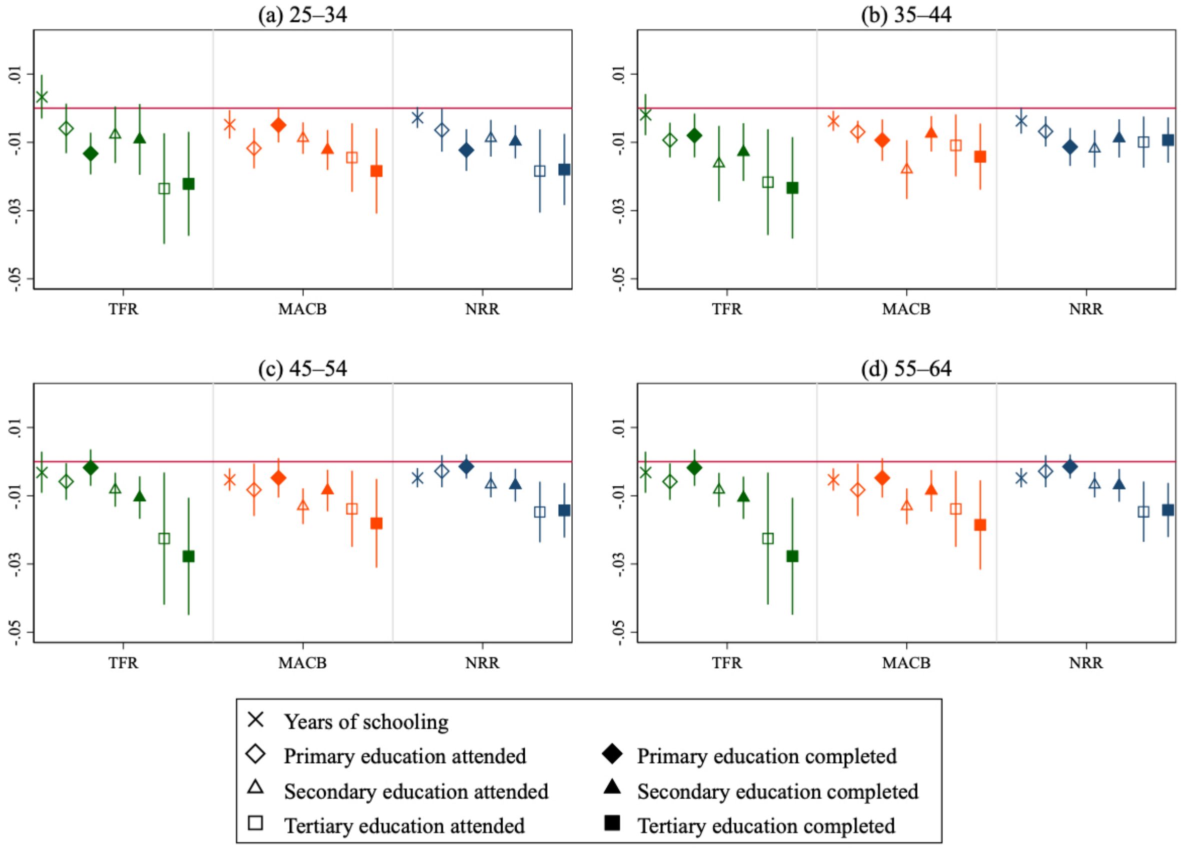

Supplement: Supplementary file 1 — Supplementary Material 1 [file 41598_2024_78735_MOESM1_ESM.pdf]
